# Molecular Phylogeny of the Bamboo Sharks (*Chiloscyllium* spp.)

**DOI:** 10.1155/2014/213896

**Published:** 2014-06-11

**Authors:** Noor Haslina Masstor, Abdullah Samat, Shukor Md Nor, Badrul Munir Md-Zain

**Affiliations:** School of Environmental and Natural Resource Sciences, Faculty of Science and Technology, Universiti Kebangsaan Malaysia, 43600 Bangi, Selangor, Malaysia

## Abstract

*Chiloscyllium*, commonly called bamboo shark, can be found inhabiting the waters of the Indo-West Pacific around East Asian countries such as Malaysia, Myanmar, Thailand, Singapore, and Indonesia. The International Union for Conservation of Nature (IUCN) Red List has categorized them as nearly threatened sharks out of their declining population status due to overexploitation. A molecular study was carried out to portray the systematic relationships within *Chiloscyllium* species using 12S rRNA and cytochrome *b* gene sequences. Maximum parsimony and Bayesian were used to reconstruct their phylogeny trees. A total of 381 bp sequences' lengths were successfully aligned in the 12S rRNA region, with 41 bp sites being parsimony-informative. In the cytochrome *b* region, a total of 1120 bp sites were aligned, with 352 parsimony-informative characters. All analyses yield phylogeny trees on which *C. indicum* has close relationships with *C. plagiosum*. *C. punctatum* is sister taxon to both *C. indicum* and *C. plagiosum* while *C. griseum* and *C. hasseltii* formed their own clade as sister taxa. These *Chiloscyllium* classifications can be supported by some morphological characters (lateral dermal ridges on the body, coloring patterns, and appearance of hypobranchials and basibranchial plate) that can clearly be used to differentiate each species.

## 1. Introduction

Over 450 sharks species with eight orders have been recognized.* Chiloscyllium*, within the family Hemiscylliidae, order Orectolobiformes, is one of the disperse populations in the ocean as their habitat extend from Indian Ocean to Western Pacific. There are seven identified species, of which only five living species have been recorded inhabiting the ocean of Malaysia* Chiloscyllium griseum *[1],* C. indicum *[2],* C. punctatum* [1],* C. plagiosum* [2], and* C. hasseltii* [3] by the Department of Fisheries Malaysia [4]. For* Chiloscyllium*, their hardiness, small size, and beautiful patterning have caused them to be commonly imported for the pet trade [5].* C. griseum*,* C. plagiosum,* and* C. punctatum* particularly are among the popular aquarium species and being kept in public aquaria [6]. Therefore, for a long time, sharks were overhunted, resulting in their globally declining populations in spite of populating wide range of water areas. Sharks are susceptible to overfishing and slow to recover if overfished, due to their low reproductive rate, late maturity, and small population size. To overcome this challenge, many conservation efforts have been introduced and applied, including listing them as IUCN Red List status. Listed as “near threatened” by the IUCN Red List [7], our study interest,* Chiloscyllium* species, has also faced decreasing population problems.

Species identification, taxonomy, and molecular systematics of shark species are among the initial steps in conserving and managing shark stocks. In spite of that, sharks phylogenetic relationships are still not well established even after 150 years of study as reported by Naylor et al. [8]. Early interest in the systematics among elasmobranchs had led to the descriptions of families of sharks and rays. Müller and Henle [9] study had established the genus* Chiloscyllium*. In 1841,* C. indicum* and two new species,* C. griseum* and* C. punctatum*, were assigned to the genus of* Chiloscyllium*. Bleeker [3] described* C. hasseltii, *which for a long time was considered a synonym of* C. griseum*. Waite [10] first placed genus* Hemiscyllium* and genus* Chiloscyllium* in their own family, Hemiscylliidae. However, Regan [11] assigned all* Chiloscyllium* species to the family Orectolobidae. Whitley [12] placed* Hemiscyllium* and* Chiloscyllium* back in family Hemiscylliidae, and this was maintained by Compagno [13]. Dingerkus and DeFino [5] identified and described two new* Chiloscyllium* species,* C. burmensis* and* C. arabicum*. Unfortunately, all of those studies were only based on the analysis of morphological data, including anatomical differences such as the neurocranium, dorsal and anal fins, visceral arch, shoulder girdle and pectoral fin, pelvic girdle and clasper, vertebrae and caudal fin, head sensory canal system, and other external morphologies [14], skeletal anatomy and dermal denticles [5], and diagnostic features and biology [6]. Those studies made a great impact on the classification of this genus, but, with the advent of molecular techniques, it can provide supportive information in various fields of study such as the molecular systematics, biogeography, population genetics, and evolutionary history [15, 16]. There are some numbers of molecular studies that had been conducted to review the phylogeny of sharks at the order and family levels [8, 17, 18] and at the species level by Human et al. [19].

Even though there were some efforts to clarify shark species relationships, as stated by Naylor et al. [8] and Heinicke et al. [20] the phylogenetic relationships among the major groups of sharks are still not well resolved. Many conflicting results were found, including the phylogenetic status of our study interest,* Chiloscyllium*. According to Goto [14],* C. indicum* is a sister taxon of all* Chiloscyllium* species, but this was contradicted by studies by Vélez-Zuazo and Agnarsson [21] and Karl et al. [22], which agreed that* C. punctatum* is the sister taxon of all* Chiloscyllium* species. Thus, within this study, we aim to clarify the phylogenetic relationships of* Chiloscyllium* species through the DNA sequencing of two mitochondrial DNA loci. The cytochrome* b *region was chosen because it is the most well-studied mtDNA gene in vertebrates [23–25], including sharks, for various taxa among elasmobranchs species [8, 26, 27], at the order level [19, 21, 28], at the family level [18, 21, 29], at the genus level [30], and at the species-specific level [31, 32]. In addition, 12S rRNA has been considered to be one conserved gene among taxa, that is, helpful for phylogenetic analysis [33]. Many researchers have agreed on the important role of phylogenetic position and evolutionary distinctiveness in establishing conservation priorities [34–36]. Information about the evolutionary history of the species and the status of their close relatives can impact conservation planning, especially for those already identified as under some level of threat [21, 37].

## 2. Materials and Methods

### 2.1. Sample Collection and DNA Tissue Extraction

**Figure 1:**
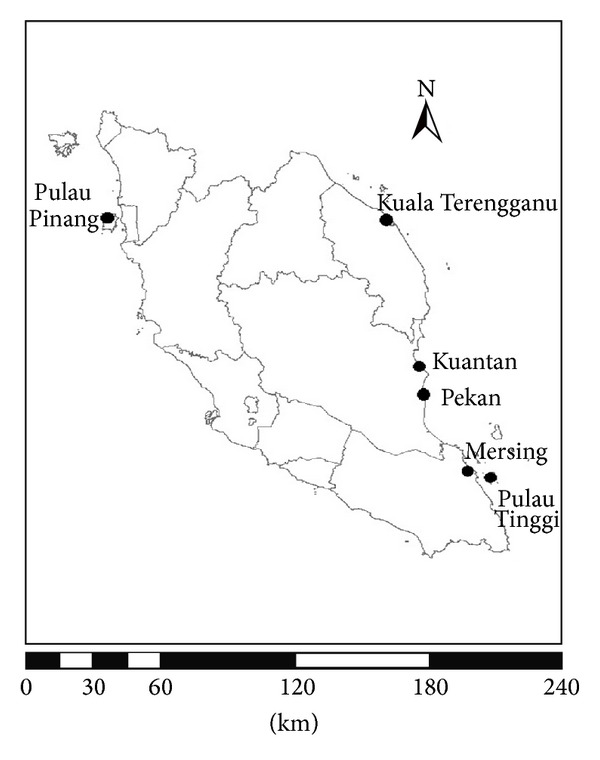
Sampling location of bamboo sharks.

Tissue samples of* C. griseum *(7),* C. indicum *(19),* C. punctatum *(4),* C. hasseltii* (4), and* Stegostoma fasciatum* (1) were obtained from freshly caught animals and preserved in 95% ethanol. All of the tissue samples were collected from fishermen in Penang, Kuala Terengganu, Kuantan, Pekan, Mersing, and Pulau Tinggi, Malaysia (Figure 1). Six DNA sequences were obtained from GenBank (Table 1). Mitochondrial DNA was isolated from 25 mg of muscle tissue using a DNeasy Tissue Kit (QIAGEN Inc.), following the manufacturer's suggested protocol.

### 2.2. DNA Amplification

A pair of elasmobranchs-specific set of primers, GluDG (5′-TGA CTT GAA RAA CCA YCG TTG-3′) and C61121H (5′-CTC CAG TCT TCG RCT TAC AAG-3′) [27], were used in the PCR for cytochrome* b *amplification. Thermal cycling for the cytochrome* b *locus consisted of an initial step at 94°C for 5 min; 30 cycles of 94°C denaturing for 30 sec, 47–50°C annealing for 30 sec, and 60°C elongation for 1 min; and 72°C post elongation for 7 min to complete the synthesis of elongated DNA molecules. The DNA extract (3 *μ*l) PCR was carried out in a final volume of 20 *μ*l reaction mixture consisting of 50–100 ng DNA template, 5 U/*μ*l Taq DNA polymerase (PROMEGA), 5X PCR buffer, 25 mM MgCl_2_, 10 mM dNTPs, and 10 pmol/*μ*l of each primer and ddH_2_O. Universal primers for the 12S rRNA gene (forward-5′-AAA CTG GGA TTA GAT ACC CCA CTA T-3′, reverse-5′-GAG GGT GAC GGG CGG TGT GT-3′) as described by Kocher et al. [38] were used with suitable modifications. Thermocycler conditions were as follows: 3 min at 95°C for initial denaturation, followed by 30 cycles of amplification (1 min at 95°C, 45 sec at 56°C, and 1 min at 72°C), and a final extension for 7 min at 72°C. Each 25 *μ*l reaction mixture contained 5 U/*μ*l Taq DNA polymerase (Vivantis), 10X PCR buffer, 50 mM MgCl_2_, 10 mM dNTPs, 10 pmol/*μ*l of each primer, 50–100 ng template DNA, and ddH_2_O. Amplified products were analyzed by electrophoresis in 1.5% agarose gel with ethidium bromide stain. PCR products were purified using a GF-1 PCR Clean-Up Kit (Vivantis), following the manufacturer's protocol. All the successfully amplified PCR products were sent to an accredited laboratory, 1st Base Laboratories Sdn Bhd, for sequencing services.

### 2.3. Data Analysis

DNA sequence editing was performed with BioEdit version 7.0.2 and visually checked. The character-based method was used to infer phylogeny for* Chiloscyllium* species. Unweighted maximum parsimony (MP) analysis was used with a heuristic search to obtain the most parsimonious MP tree. This phylogenetic analysis was conducted using Phylogeny Analysis Using Parsimony (PAUP) version 4.0 [39]. Bootstrap analysis was performed using 1000 replications to assess robustness of branching patterns and analysis reliability.

Model_test 3.7 [40] was used to choose the substitution model that best fits the data using the Akaike information criterion (AIC) for Bayesian analysis. Bayesian analyses were conducted using MrBayes 3.1 for both 12S rRNA and cytochrome* b* regions. The most appropriate model for 12S rRNA was found to be TrN+I (−lnL = 1131.1022; AIC = 2274.2043). The estimated base frequencies were as follows: A, 0.3303; C, 0.2764; G, 0.1937; and T, 0.1997. Estimated substitution rates among these nucleotides were 1.0000 for A/C, A/T, C/G, and G/T; 2.4579 for A/G; and 5.9223 for C/T and the estimated proportion of invariable site was 0.6192 with equal rates. Bayesian inference was made by running two simultaneous metropolis-coupled MCMC chains for 380,000 generations with 0.009887 split frequency probability. Trees were sampled every 100 generations and a consensus tree was generated from 2850 trees by omitting the first 950 trees. For the cytochrome* b* sequences, the best model computed for Bayesian analysis was TrN+I (−lnL = 5441.2451; AIC = 10894.4902). The base frequency estimates were as follows: A, 0.3087; C, 0.2734; G, 0.1203; and T, 0.2976. Estimated substitution rates among these nucleotides were 1.0000 for A/C, A/T, C/G, and G/T; 2.6360 for A/G; and 4.7907 for C/T. Bayesian inferences were run for 550,000 generations (0.009769 split frequency probability) and a consensus tree was constructed from 4125 trees with 1375 burn-in.

12S rRNA and cytochrome* b* data sets were combined together to construct one consensus tree. The incongruence-length difference (ILD)/partition homogeneity test was conducted to test the partition incongruence to overcome the issue of combining those two data sets [41]. These combined data sets were analyzed for MP analysis with heuristic search and 1000 replications of bootstrap analysis, using PAUP version 4.0 [39] and Bayesian inference (BI) under Akaike information criterion (AIC) framework. A nucleotide substitution model, TrN+I (−lnL = 6263.0845; AIC = 12538.1689), was selected using Model_test 3.7 [40] for BI with estimated base frequency as follows: A = 0.3104; C = 0.2740; G = 0.1389; T = 0.2768 and estimated substitution rates as follows: 1.0000 for A/C, A/T, C/G, and G/T; 2.1965 for A/G; and 4.2050 for C/T, and proportion of invariable sites (I) was 0.4677 with equal rates. A consensus tree was constructed from 1575 trees and 525 burn-in through 210,000 generations (trees were sampled every 100 generations) and 0.008094 split frequency probability.

## 3. Results 

### 3.1. Data Analyses and Phylogenetic Resolution

#### 3.1.1. Maximum Parsimony (MP)

**Figure 2:**
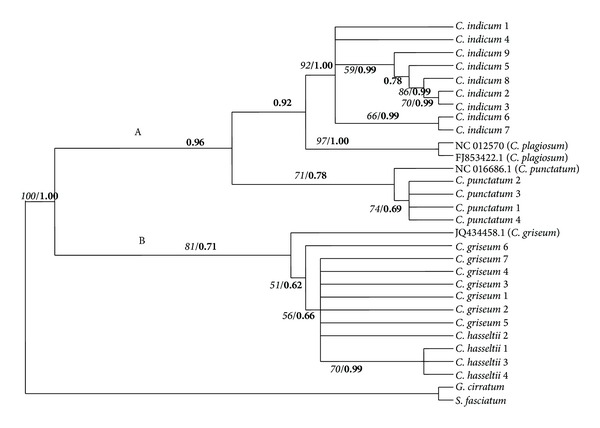
Maximum parsimony cladogram of partial 12S rRNA gene sequences and Bayesian posterior probability tree. Italic numbers at the branches stand for bootstrap values higher than 50% of 1000 replications and bold numbers stand for Bayesian posterior probability value.

A total of 381 characters were sequenced for the 12S rRNA region; 302 were constant characters, 38 variable characters were parsimony-uninformative, and 41 characters were parsimony-informative. Maximum parsimony analysis resulted in one most parsimonious tree (Figure 2) with 109 steps, consistency index (CI) = 0.8349, homoplasy index (HI) = 0.1651, and retention index (RI) = 0.9122 for 12S rRNA sequences. Two distinct branches divided* Chiloscyllium* species into two main clades: Clade A, consisting of* C. indicum*,* C. plagiosum*, and* C. punctatum* individuals, and Clade B, consisting of* C. griseum* and* C. hasseltii* individuals.* C. indicum* formed its own clade, with 92% bootstrap support value. With 97% bootstrap support,* C. plagiosum* became a sister taxon of* C. indicum*.* C. punctatum* individuals were grouped into their own clade with 71% bootstrap support and became a sister taxon to* C. indicum* and* C. plagiosum*. The three individuals of* C. hasseltii* (*C. hasseltii* 1,* C. hasseltii* 3, and* C. hasseltii* 4) formed a clade with 70% bootstrap support. However, one* C. hasseltii* sample (*C*.* hasseltii* 2) fell into the polytomy of* C. griseum* group, forming a nonmonophyletic clade with 81% bootstrap support.

**Figure 3:**
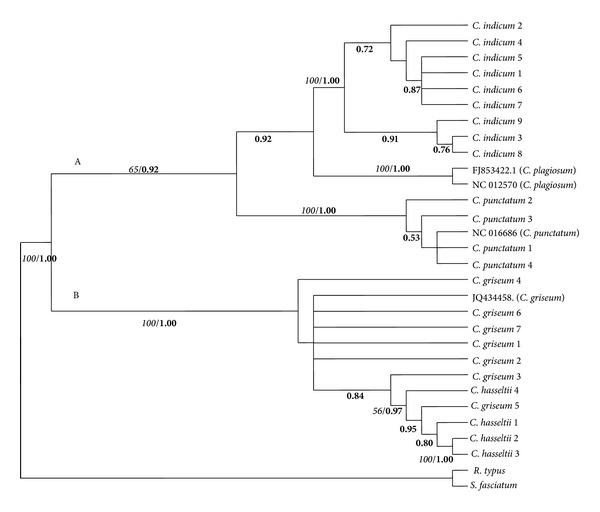
Maximum parsimony cladogram of partial cytochrome* b* gene sequences and Bayesian posterior probability tree. Italic numbers at the branches stand for bootstrap values higher than 50% of 1000 replications and bold numbers stand for Bayesian posterior probability value.

For the cytochrome* b* region, a total of 1120 characters were aligned in MP analysis; 596 characters were constant, 172 variable characters were parsimony-uninformative, and 352 characters were parsimony-informative. One most parsimonious tree was recovered, with 893 tree length, CI = 0.7436, HI = 0.2564, and RI = 0.8842 (Figure 3). As with the 12S rRNA tree topology,* Chiloscyllium* species was divided into two different clades: Clade A, consisting of* C. indicum*,* C*.* plagiosum*, and* C. punctatum*, and Clade B, consisting of* C. griseum* and* C. hasseltii*, with 65% and 100% bootstrap support, respectively.* C. plagiosum* and* C. indicum* formed their own clade, both with 100% bootstrap support, and became sister taxa of each other.* C. punctatum* also formed its own clade, with 100% bootstrap support. In another distinct clade (*C. griseum* and* C. hasseltii*), most* C. griseum* individuals displayed polytomies, while* C. hasseltii* created its own clade. The* C. griseum* and* C. hasseltii* clade was not monophyletic, as there were two* C. griseum* individuals in the* C. hasseltii* group. Clade B is a sister taxon of Clade A.

### 3.2. Bayesian Analysis

Bayesian analysis for 12S rRNA data set produced essentially the same topology as the MP analysis (Figure 2) and revealed a separation of two branches, with Bayesian posterior probabilities of 0.96 for a* C. indicum*,* C. plagiosum*, and* C. punctatum *group (Clade A) and 0.71 for a* C. griseum* and* C*.* hasseltii* group (Clade B). A 1.00 Bayesian posterior probability supports the clustering of the* C. punctatum* branch.* C. indicum* and* C. plagiosum* became a sister clade with 0.92 Bayesian posterior probability support. Like 12S rRNA, cytochrome* b *Bayesian tree topology also formed two distinct clades (Figure 3): Clade A, consisting of* C. indicum, C. plagiosum*, and* C. punctatum* individuals, and Clade B, consisting of* C. griseum* and* C. hasseltii* individuals, with 0.92 and 1.00 Bayesian posterior probability support, respectively. A Bayesian posterior probability of 0.92 supports the clustering of the* C. indicum* and* C. plagiosum* clade.* C. punctatum* individuals formed their own clade, with 1.00 Bayesian posterior probability.

### 3.3. Consensus Analysis

**Figure 4:**
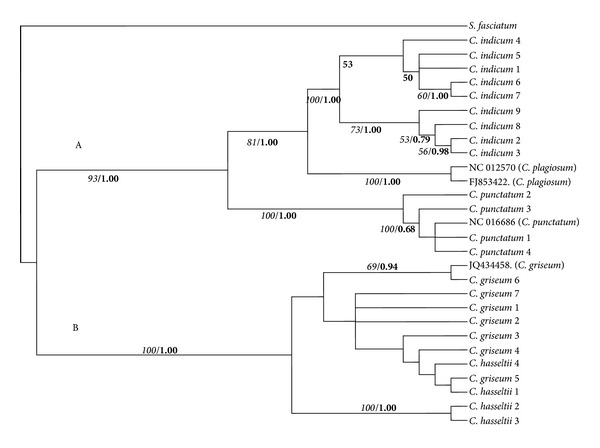
Inferred phylogeny of* Chiloscyllium* species, a majority rule consensus tree based on maximum parsimony and Bayesian analysis. Italic numbers at the branches stand for bootstrap values higher than 50% of 1000 replications and bold numbers stand for Bayesian posterior probability value.

ILD test simulated nonsignificant *P* value > 0.05 (0.5200), meaning that 12S rRNA and cytochrome* b* analyses should be proceeded for phylogenetic reconstruction. The MP analysis based on equally weighted total substitutions for combined data of 12S rRNA and cytochrome* b* sequences produced a tree with 893 steps, CI = 0.7828, HI = 0.2172, and RI = 0.9038. Out of 1501 characters sequenced for both regions, 32 characters were constant, 232 variable characters were parsimony-uninformative, and 337 were parsimony-informative. The results of the MP and Bayesian analysis of all data combined are shown in Figure 4. These results were broadly similar to all analysis for the 12S rRNA and cytochrome* b* regions.* Chiloscyllium* species was divided into two major sister clades that clustered* C. griseum* and* C. hasseltii* in one group (Clade B) and* C. indicum*,* C. plagiosum*, and* C. punctatum *clade in the other (Clade A). The* C. griseum* and* C. hasseltii* nonmonophyletic clade was supported with 100% bootstrap and 1.00 Bayesian posterior probability.* C. indicum, C. plagiosum, *and* C. punctatum* clustered into their own clade, with 100% bootstrap support and 1.00 Bayesian posterior probability.* C. indicum* and* C. plagiosum* grouped together, with 81% bootstrap support and 1.00 Bayesian posterior probability.* C. punctatum* became sister taxon of these two species, with 93% bootstrap support and 1.00 Bayesian posterior probability.

## 4. Discussion

This molecular study has led to a better understanding of the relationships among* Chiloscyllium*. Tree topologies of the 12S rRNA region show similarities to the cytochrome* b* tree topologies for all of the analyses conducted.* C. indicum* formed its own monophyletic group and became a sister taxon of* C. plagiosum*, while* C. punctatum *group became a sister taxon of the* C. indicum* and* C. plagiosum* clade. The nonmonophyly within* C. griseum* and* C. hasseltii *was observed using the two different molecular markers for all analyses. However, it can be differentiated into two groups: (1)* C. griseum* group and (2)* C. hasseltii* group. Such grouping actually reflects the close relationships between these two species. Vélez-Zuazo and Agnarsson [21] and Naylor et al. [42] also indicated these two species as sister species. According to Dingerkus and DeFino [5],* C. hasseltii* adults look almost identical to* C. griseum* adults; however, they can still be distinguished by morphometric measurements. For* C. griseum*, the distance between the first and second dorsal fins is usually more than 9.3% of total length (less than 9.3% in* C. hasseltii*); the height of the first dorsal fin is usually more than 6.6% of total length (less than 6.6% in* C. hasseltii*); and the height of the second dorsal fin is usually more than 5.8% of total length (less than 5.8% in* C. hasseltii*).* C. hasseltii* juveniles are separable from* C. griseum* by having black bands outlined pattern, whereas* C. griseum* does not have the bands outlined in black [5]. Therefore, our nonmonophyly* C. griseum*/*C. hasseltii* groups might occur due to some missing data and uneven taxon sampling which affect our topology inadequacies. Furthermore, according to Heist [43], coastal and benthic sharks like* C. griseum* and* C. hasseltii* exhibit only a little divergence in nuclear and mitochondrial gene frequencies where they are continuously distributed along continental margins.

All of the research results showed that* Chiloscyllium* species is separated into two distinctive clades: clade* C. griseum* and* C. hasseltii* (Clade B) and its sister clade, the* C. indicum, C. plagiosum,* and* C. punctatum* clade (Clade A).* C. punctatum* is a sister taxon of* C. indicum* and* C. plagiosum*. This shows that* C. indicum* is having the closest genetic relationships with* C. plagiosum* while their close sister is* C. punctatum*.* C. griseum* is the close sister to* C. hasseltii*. This finding is contrary to the study conducted by Vélez-Zuazo and Agnarsson [21], which identified* C. indicum* and* C. plagiosum* as sisters,* C. griseum* and* C. hasseltii* as sisters, and* C. punctatum* as sister of all the other* Chiloscyllium* species. However, Vélez-Zuazo and Agnarsson's [21] suggested topology was questionable because the authors themselves noted that their analysis suffered from a substantial amount of missing data (85%) and their data came directly from GenBank [42] which might bear some errors and inaccuracies. Karl et al. [22] proposed that* C. indicum* is a sister of* C. plagiosum*,* C. hasseltii* is a sister of those two species, and* C. punctatum* is a sister of all the* Chiloscyllium* species while Goto [14] placed* C. indicum* as a sister species of all the other* Chiloscyllium *species based on their morphological synapomorphies in mode of reproduction, maximum number of offspring, and existences of vestibular horns or silky fibers on egg case. Our molecular results agreed with the hypothesis suggested by Naylor et al. [42] inferred from a Bayesian analysis based on NADH2 sequence data.* C. indicum* and* C. plagiosum* are sisters to each other and their close species is* C. punctatum*, while* C. griseum* and* C. hasseltii* formed their own respective clade. The phylogenetic placement of the specimen of* Hemiscyllium ocellatum* was found to be model dependent [42]. Dingerkus and DeFino [5] cladogram based on morphological characters was also supported by our study hypothesis. According to Dingerkus and DeFino [5], genus* Chiloscyllium* can be defined as monophyletic by possessing less than 180 vertebral, strong middorsal ridges that extend from the back of the head onto the tail, fourth and fifth gill openings that meet dorsally and ventrally, and a projection on the posterior border of the spiracular opening, pointing into the spiracle. Dingerkus and DeFino [5] indicated that* C. plagiosum* and* C. indicum* are sister species by sharing the following morphology: lateral dermal ridges on the body; specially modified dermal denticles on these dermal ridges; dermal denticles covering the rest of the body; and gill arches that are of the primitive* Chiloscyllium* type, except that* C. indicum* has lost the small basibranchial element. Dingerkus and DeFino [5] also positioned* C. punctatum* as the closest species to the* C. plagiosum* and* C. indicum* sister-species clade. It is followed by* C. griseum*,* C. hasseltii*, and two new species,* C. confusum *and* C. burmensis*. These remaining species form a group based on the three hypobranchial elements projecting backward to almost (or fully) touch the basibranchial plate, the basibranchial element being greatly reduced (or absent) and the adults being uniform in color dorsally, having lost any juvenile color or patterns [5]. The* C. hasseltii*,* C. griseum*,* C. confusum*, and* C. burmensis* group is defined by having the third hypobranchials articulating with the basibranchial plate, the absence of basibranchial elements, and dermal denticles as broad as they are long. This indicates that* C. griseum* and* C. hasseltii* shared some common characteristics that placed them as sister-species taxa, as shown by the present findings.* C. hasseltii* is the sister group of* C. confusum* and* C. burmensis,* based on the presence of mid-lateral keels on the dermal denticles [5].

## 5. Conclusion 

Our study indicated that the cytochrome* b *and 12S rRNA genes are reliable tools to investigate the genetic relationships among five species of bamboo sharks. This study could be improved by increasing taxon sampling and adding study regions from nuclear genes to gain more informative phylogenetic estimation. These results should be considered as initial step toward understanding* Chiloscyllium* relationships as they were inferred analysis from two fast-evolving mitochondrial genes. This deposition of the* Chiloscyllium* species will pave the way for future discussion on shark evolution, conservation strategies, fisheries management, and benefit as a preliminary reference toward a better conservation assessment.

## Figures and Tables

**Table 1:** Details of the samples included in the study.

Number	Species	Species code	Common name	Type of sample	Places of sampling
1	*C. indicum *	*C. indicum* 1	Slender bamboo shark	Tissue	Mersing, Johor
2	*C. indicum *	*C. indicum* 2	Slender bamboo shark	Tissue	Mersing, Johor
3	*C. indicum *	*C. indicum* 3	Slender bamboo shark	Tissue	Mersing, Johor
4	*C. indicum *	*C. indicum* 4	Slender bamboo shark	Tissue	Mersing, Johor
5	*C. griseum *	*C. griseum* 1	Grey bamboo shark	Tissue	Mersing, Johor
6	*C. griseum *	*C. griseum* 2	Grey bamboo shark	Tissue	Mersing, Johor
7	*C. indicum *	*C. indicum* 5	Slender bamboo shark	Tissue	Pulau Tinggi, Johor
8	*C. indicum *	*C. indicum* 6	Slender bamboo shark	Tissue	Pulau Tinggi, Johor
9	*C. indicum *	*C. indicum* 7	Slender bamboo shark	Tissue	Pulau Tinggi, Johor
10	*C. punctatum *	*C. punctatum* 1	Grey carpet shark	Tissue	Pulau Tinggi, Johor
11	*C. hasseltii *	*C. hasseltii* 1	Indonesian bamboo shark	Tissue	Pulau Pinang
12	*C. punctatum *	*C. punctatum* 2	Grey carpet shark	Tissue	Pulau Pinang
13	*C. indicum *	*C. indicum* 8	Slender bamboo shark	Tissue	Pulau Pinang
14	*C. indicum *	*C. indicum* 9	Slender bamboo shark	Tissue	Pulau Pinang
15	*C. hasseltii *	*C. hasseltii* 2	Indonesian bamboo shark	Tissue	Pulau Pinang
16	*C. hasseltii *	*C. hasseltii* 3	Indonesian bamboo shark	Tissue	Pulau Pinang
17	*C. hasseltii *	*C. hasseltii* 4	Indonesian bamboo shark	Tissue	Pulau Pinang
18	*C. punctatum *	*C. punctatum* 3	Grey carpet shark	Tissue	Mersing, Johor
19	*C. griseum *	*C. griseum* 3	Grey bamboo shark	Tissue	Kuantan, Pahang
20	*C. punctatum *	*C. punctatum* 4	Grey carpet shark	Tissue	Kuantan, Pahang
21	*C. griseum *	*C. griseum* 4	Grey bamboo shark	Tissue	Kuantan, Pahang
22	*C. griseum *	*C. griseum* 5	Grey bamboo shark	Tissue	K. Terengganu, Terengganu
23	*C. griseum *	*C. griseum* 6	Grey bamboo shark	Tissue	Pekan, Pahang
24	*C. griseum *	*C. griseum* 7	Grey bamboo shark	Tissue	Pekan, Pahang
25	*S. fasciatum *		Zebra shark	Tissue	Pekan, Pahang
26	*G. cirratum *	AY830745.1	Nurse shark		GenBank
27	*R. typus *	AM265573.1	Whale shark		GenBank
28	*C. griseum *	JQ434458.1	Grey bamboo shark		GenBank
29	*C. punctatum *	NC_016686.1	Grey carpet shark		GenBank
30	*C. plagiosum *	FJ853422.1	Whitespotted bamboo shark		GenBank
31	*C. plagiosum *	NC_012570.1	Whitespotted bamboo shark		GenBank
